# Histopathology Is Key to Interpreting Multiplex Molecular Test Results From Postmortem Minimally Invasive Tissue Samples

**DOI:** 10.1093/cid/ciab772

**Published:** 2021-12-15

**Authors:** Jana M Ritter, Josilene N Seixas, Edwin Walong, Jeanette Dawa, Clayton Onyango, Fabiana C Pimenta, Maria da Gloria Carvalho, Luciana Silva-Flannery, Tiffany Jenkinson, Katie Howard, Julu Bhatnagar, Maureen Diaz, Jonas M Winchell, Sherif R Zaki, Sandra S Chaves, Roosecelis B Martines

**Affiliations:** 1 Division of High-Consequence Pathogens and Pathology, Centers for Disease Control and Prevention, Atlanta, Georgia, USA; 2 College of Health Sciences, University of Nairobi, Nairobi, Kenya; 3 Washington State University, Global Health Programs (Kenya office), Nairobi, Kenya; 4 Division of Global Health Protection, Centers for Disease Control and Prevention, Nairobi, Kenya; 5 Division of Bacterial Diseases, Centers for Disease Control and Prevention, Atlanta, Georgia, USA; 6 Influenza Program, Centers for Disease Control and Prevention, Nairobi, Kenya and Influenza Division, Centers for Disease Control and Prevention, Atlanta, Georgia, USA

**Keywords:** minimally invasive tissue sampling, MITS, multiplex PCR, TAC, TaqMan® array card, histopathology

## Abstract

**Background:**

Minimally invasive tissue sampling (MITS) is an alternative to complete autopsy for determining causes of death. Multiplex molecular testing performed on MITS specimens poses challenges of interpretation, due to high sensitivity and indiscriminate detection of pathogenic, commensal, or contaminating microorganisms.

**Methods:**

MITS was performed on 20 deceased children with respiratory illness, at 10 timepoints up to 88 hours postmortem. Samples were evaluated by multiplex molecular testing on fresh tissues by TaqMan® Array Card (TAC) and by histopathology, special stains, immunohistochemistry (IHC), and molecular testing (PCR) on formalin-fixed, paraffin-embedded (FFPE) tissues. Results were correlated to determine overall pathologic and etiologic diagnoses and to guide interpretation of TAC results.

**Results:**

MITS specimens collected up to 3 days postmortem were adequate for histopathologic evaluation and testing. Seven different etiologic agents were detected by TAC in 10 cases. Three cases had etiologic agents detected by FFPE or other methods and not TAC; 2 were agents not present on TAC, and 2 were streptococci that may have been species other than those present on TAC. Result agreement was 43% for TAC and IHC or PCR, and 69% for IHC and PCR. Extraneous TAC results were common, especially when aspiration was present.

**Conclusions:**

TAC can be performed on MITS up to 3 days after death with refrigeration and provides a sensitive method for detection of pathogens but requires careful interpretation in the context of clinicoepidemiologic and histopathologic findings. Interpretation of all diagnostic tests in aggregate to establish overall case diagnoses maximizes the utility of TAC in MITS.

Mortality surveillance is critical to improving health outcomes and reducing mortality rates in low- and middle- income countries (LMICs) [1, 2]. Complete diagnostic autopsy (CDA) is the gold standard for cause of death determination but is not routinely performed, especially in LMICs [3–6]. In settings where verbal autopsy is conducted, its sensitivity in identifying infectious disease and agreement with CDA is low [6, 7]. Minimally invasive tissue sampling (MITS) approaches utilizing postmortem needle biopsies, in conjunction with verbal autopsy, are gaining acceptance as an alternative to CDA [4, 6, 8].

MITS specimens can be used for histopathologic evaluation and various microbiological test methods, although results from MITS studies have shown variable concordance with CDA [6, 9, 10]. A recent study conducted at Kenyatta National Hospital showed an overall histologic concordance rate of 66% and pathogen detection using multiplex molecular testing concordance rate of 80% between specimens collected using MITS techniques and CDA [11]. MITS is now increasingly being used in combination with laboratory tests for cause of death determination in low-resource settings [2, 6]. Nonetheless, interpretation of laboratory results performed on MITS specimens can be challenging, and even more so when utilizing multiplex assays that detect a wide range of infectious agents that could represent any combination of pathogenic, commensal, or contaminating microorganisms, the presence of which could be influenced by postmortem interval [12-15].

Here we evaluate effects of postmortem interval on histopathology and detection of infectious agents and describe a process for interpreting TaqMan® Array Card (TAC) results from MITS samples. As described by Dawa et al, we demonstrated reliability of TAC on MITS specimens obtained up to 88 hours after death and that concordance of TAC results varied within and among tissue types from the same patient [16]. Herein, we describe the process of interpreting the significance of TAC results in the context of disease etiology and highlight the importance of correlating TAC and other laboratory results with pathologic findings when assessing the contribution of specific infectious agents to causes of death.

## METHODS

Study design, sample collection, and molecular testing by TAC have been described in detail [13, 16]. The list of infectious agents tested by TAC is provided in Supplementary Table 1. In brief, 20 deceased children (median age, 8 months [range, 1–53 months]) with hospital diagnoses of respiratory illness were enrolled. Refrigerated bodies were sampled immediately and then every 6 hours for 9 collections, followed by a final collection 24 hours later. All cases had lung and liver specimens collected for histopathology and lung specimens collected for TAC; 5 cases also had blood and liver specimens collected for TAC testing. A list of agents tested by TAC and detailed methods for histology, immunohistochemistry (IHC), polymerase chain reaction (PCR) on formalin-fixed, paraffin-embedded (FFPE) MITS specimens, and assessment of MITS quality by histopathology are provided in the Supplementary Materials.

### Assessing TAC Result Significance and Achieving Case Diagnoses

**Figure 1.**
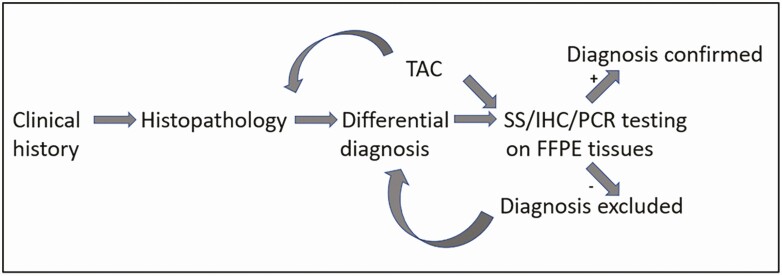
Iterative process for achieving etiologic diagnoses in minimally invasive tissue sampling specimens. Abbreviations: FFPE, formalin-fixed, paraffin-embedded; IHC, immunohistochemistry; PCR, polymerase chain reaction; SS, special stains; TAC, TaqMan Array Card.

TAC results were interpreted as positive or negative based on cycle threshold cutoff value of *≤*40 with result validation as established during assay development and optimization [13]. Positive TAC results were considered “contributory” if histopathologic evidence of disease attributable to the agent was present and the agent was confirmed by IHC or PCR testing, as available. TAC results were considered “equivocal” when consistently detected (>4 of 10 timepoints) but without corresponding histopathology or detection by other methods, or when there exists the possibility of the agent’s significance without overt pathology (eg, acute sepsis or viremia). TAC results were considered “extraneous” when characteristic histopathology was absent and the agent was detected only in a few (<4 of 10) specimens by TAC, or if aspiration was present and the agent could be reasonably attributed to that. Aspiration was diagnosed based on presence of foreign material (eg, food, squames) with or without mixed bacteria, and with or without associated granulomatous inflammatory response. Individual overall case diagnoses were achieved in an iterative process, taking into consideration available clinical history, histopathologic findings, and test results (Figure 1). Correlation of histopathology, TAC, and other test methods was performed across all cases to assess the utility and contribution of each to case diagnosis in MITS.

### Ethical Considerations

Ethical approval to conduct the study was provided by the Kenya Medical Research Institute, Scientific Ethics Review Unit (KEMRI/SERU/CGHR/1003562) and the Kenyatta National Hospital–University of Nairobi Ethics Review Committee (P82/02/2018).

## RESULTS

### MITS Adequacy and Quality

**Figure 2.**
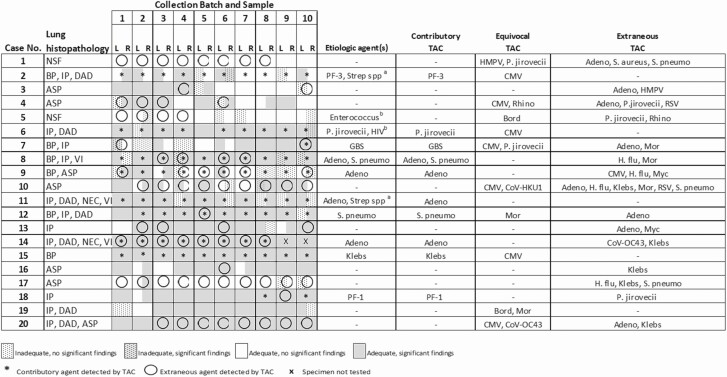
Pathologic findings and detection of pathogens by TaqMan Array Card (TAC) in serial left lung minimally invasive tissue sampling specimens up to 72 hours after death for 20 cases. Overall significant histopathologic findings identified in lung, etiologic agents assigned, categorization of agents detected by TAC, and presence or absence of pathology and contributory or extraneous TAC results at each collection timepoint. Postmortem interval for batches (hours): 1: 7–17; 2: 13–22; 3: 18–28; 4: 24–34; 5: 31–40; 6: 37–47; 7: 47–52; 8: 48–58; 9: 55–64; 10: 77–88. ^a^*Streptococcus* spp may not be detected by lung TAC. ^b^Agent detected in liver or blood and not present on lung TAC. Abbreviations: Adeno, adenovirus; ASP, aspiration; Bord, *Bordetella*; BP, bronchopneumonia; CMV, cytomegalovirus; CoV, coronavirus; DAD, diffuse alveolar damage; GBS, group B *Streptococcus*; H. flu, *Haemophilus influenzae*; HIV, human immunodeficiency virus; HMPV, human metapneumovirus; IP, interstitial pneumonitis; Klebs, *Klebsiella*; L, left; Mor, *Moraxella*; Myc, *Mycoplasma*; NEC, necrosis; NSF, no significant findings; PF-1, parainfluenza virus 1; PF-3, parainfluenza virus 3; P. jirovecii, *Pneumocystis jirovecii*; R, right; Rhino, rhinovirus; RSV, respiratory syncytial virus; S. aureus, *Staphylococcus aureus*; S. pneumo, *Streptococcus pneumoniae*; Strep, *Streptococcus*; TAC, TaqMan Array Card; VI, viral inclusions.

Overall technical adequacy of tissue for histopathologic evaluation was 92% (184/200) for right lung, 87% (174/200) for left lung, and 99% (99/100) for liver specimens (Figure 2). Seven of 43 (16%) technically inadequate specimens had significant histopathologic findings in the small fragments of target tissue present. Nontarget tissue was present in 49 of 200 (25%) right lung, 74 of 200 (37%) left lung, and 11 of 200 (6%) liver specimens. Nontarget tissue present with lung specimens included muscle, heart, connective tissue, airway, liver, fat, bone, marrow, vessel, cartilage, thymus, lymph node, and skin. Nontarget tissue present with liver specimens included muscle, gastrointestinal tissue, kidney, lung, skin, and connective tissue.

Autolysis was minimal up to the last sample collections (77–88 hours after death) for all cases and did not interfere with assessment of tissue morphology or pathology. There was also no evidence of postmortem bacterial overgrowth by examination of hematoxylin and eosin– or Gram-stained tissue sections. Instead, all bacteria seen were attributable to pathologic infectious disease processes (n = 3), aspiration (n = 1), or contamination from gastrointestinal tissue (n = 1).

### Effect of Postmortem Interval on Histopathologic Findings

One hundred twenty-six of 200 (63%) left lung and 129 of 200 (65%) right lung specimens from 18 of 20 (90%) cases had significant histopathology (Figure 2). These included bronchopneumonia in 6 of 20 (30%), interstitial pneumonitis in 11 of 20 (55%), diffuse alveolar damage in 7 of 20 (35%), necrosis in 2 of 20 (10%), viral inclusions in 3 of 20 (15%), and acute and chronic aspiration in 7 of 20 (35%) cases, with >1 finding present in 10 of 20 (50%) cases. Cases with significant infectious etiologies identified in the lung (n = 10) had a higher number of lung samples (mean 16, median 19 of 20 total lung samples) with significant histopathologic findings than cases without infectious etiologies identified in the lung (n = 10; mean 11, median 12). There was no significant difference among number of collection timepoints (range 23–28, median 25/40) for presence of diagnostic lesions in lung specimens, and no particular timepoint or range was better for identifying lung lesions. Notable findings in liver tissues were limited to hepatocellular necrosis in 3 cases, steatosis compatible with malnutrition in 2 cases, and presence of bacteria in 2 cases with sepsis. One sepsis case (case number 15) also had bacterial bronchopneumonia, and the other was identified only in the liver specimens (case number 5). Liver findings were consistent across all 10 timepoints of sample collection for each case. Since lung pathology predominated among these cases, further detailed analysis was limited to lung specimens.

### Agreement of TAC With Histopathology and Other Test Methods

Etiologic agents (ie, those deemed significant to the observed pathology and to the patient’s cause of death) identified for each case and associated contributory, equivocal, and extraneous TAC results are shown in Figure 2. Seven different etiologic agents present on the lung TAC (parainfluenza viruses 1 and 3, *Pneumocystis jirovecii*, *Streptococcus pneumoniae*, group B *Streptococcus*, adenovirus, and *Klebsiella* spp) were identified 11 times among 10 cases. *Streptococcus* spp was identified by IHC in 2 cases with negative TAC results for the streptococci on the card (*S. pneumoniae* and group A and group B streptococci). The antibody utilized in the IHC assay against *S. pneumoniae* is known to cross-react with other streptococci not tested by the TAC. Therefore, these cases likely had nonpneumococcal, non–group A, non–group B streptococcal infection. Two additional agents not present on the TAC (human immunodeficiency virus [HIV] and *Enterococcus*) were identified by other test methods in nonpulmonary specimens from 2 cases.

Of the 11 cases for which TAC detected an etiologic agent, the agent was detected by TAC in an average of 7 of 10 (median, 9) timepoints per agent per case. TAC results deemed extraneous were obtained for 13 different agents, a total of 35 times in 15 cases, and an average of 3 of 10 (median 1) timepoints per agent per case. Contributory and extraneous TAC results were detected together at 20 timepoints among 5 cases. Cytomegalovirus (CMV) was the most frequent equivocal TAC result, detected in 7 cases and an average of 8 of 10 (median, 9) timepoints per case.

Correlation of significance of histopathologic findings with significance of TAC results was determined. Significant histopathologic findings were present in at least 1 (right or left) lung specimen at 53 of 59 (90%) timepoints with contributory TAC results only, 16 of 20 (80%) timepoints with both contributory and extraneous TAC results, and only 28 of 52 (54%) timepoints with extraneous TAC results only. Overall agreement was 29% for TAC and IHC results, 17% for TAC and PCR, 43% for TAC and either method (IHC or PCR), and 69% for IHC and PCR (Supplementary Table 3). Case-level agreement for the various test methods are shown by infectious agent in Table 1; agreement was higher for IHC and PCR than for TAC and either method for all agents, except for *S. pneumoniae*, group A *Streptococcus*, and group B *Streptococcus*.

**Table 1.** Case-Level Agreement of TaqMan Array Card (TAC), Immunohistochemistry, and Polymerase Chain Reaction Test Results, by Agent Detected on TAC for 20 Cases

	No. (%) of Cases With Agreement			
Agent	TAC/IHC	TAC/PCR	IHC/PCR	TAC/IHC or PCR
Adenovirus	4/12 (33)	3/9 (27)	11/12 (91)	4/12 (36)
*Bordetella*	0/2 (0)	…	…	…
CMV	0/8 (0)	0/8 (0)	8/8 (100)	0/8 (0)
*Haemophilus*	0/4 (0)	…	…	…
HMPV	0/2 (0)	1/2 (50)	1/2 (50)	1/2 (50)
*Klebsiella*	3/6 (50)	…	…	…
*Mycoplasma*	…	0/2 (0)	…	…
Parainfluenza 1	0/1 (0)	0/1 (0)	1/1 (100)	0/1 (0)
Parainfluenza 3	1/1 (100)	1/1 (100)	1/1 (100)	1/1 (100)
*Pneumocystis* ^a^	1/6 (17)	…	…	…
Rhinovirus	…	0/2 (0)	…	…
RSV	0/2 (0)	0/2 (0)	2/2 (100)	0/2 (0)
*Streptococcus pneumoniae*	4/5 (80)	1/5 (20)	2/5 (40)	4/5 (80)
Group B *Streptococcus*	1/1 (100)	…	…	…
Group A *Streptococcus*	1/1 (100)	0/1 (0)	0/1 (0)	1/1 (100)
*Staphylococcus aureus*	0/1 (0)	…	…	…

Abbreviations: CMV, cytomegalovirus; HMPV, human metapneumovirus; IHC, immunohistochemistry; PCR, polymerase chain reaction; RSV, respiratory syncytial virus; TAC, TaqMan Array Card.

^a^For purposes of correlating test results, Grocott Methenamine Silver stain results for *Pneumocystis* were considered equivalent to IHC results for other agents.

**Figure 3.**
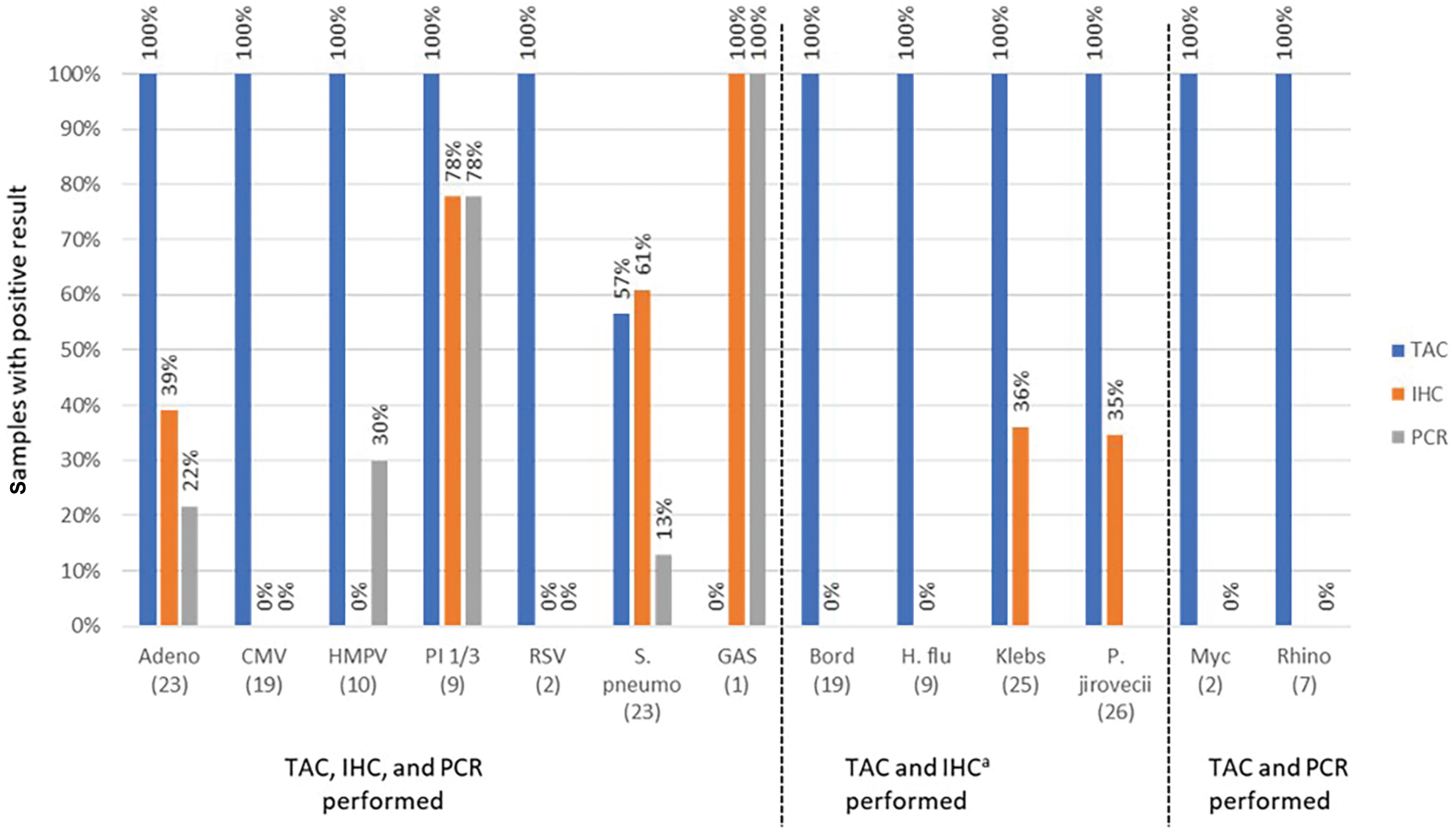
Detection of agents by various test methods in samples from individual minimally invasive tissue sampling collections, without regard to assessment of agent significance (n = number of samples with testing performed by all available methods). TaqMan Array Card (TAC), immunohistochemistry (IHC), and polymerase chain reaction (PCR) were performed for adenovirus (Adeno), cytomegalovirus (CMV), human metapneumovirus (HMPV), parainfluenza virus (PI) 1/3, respiratory syncytial virus (RSV), *Streptococcus pneumoniae* (*S. pneumoniae*), and group A *Streptococcus* (GAS). TAC and IHC were performed for *Bordetella* (Bord), *Haemophilus influenzae* (H. flu), *Klebsiella* (Klebs), and *Pneumocystis jirovecii* (P jirovecii). TAC and PCR were performed for *Mycoplasma* (Myc) and rhinovirus (Rhino). ^a^For purposes of correlating tests results, Grocott Methenamine Silver stain results for *Pneumocystis* were considered equivalent to IHC results for other agents.

In addition to determining case-level agreement for the various test methods employed, comparison of agent detection by each test method for individual specimens was performed. Only samples from timepoints with all available test methods performed were considered for this comparison (Figure 3). TAC had more frequent detection than other methods for all agents except *S. pneumoniae* and was often the only method by which an agent was detected.

### Determination of Etiologic Diagnoses and TAC Contribution

**Figure 4.**
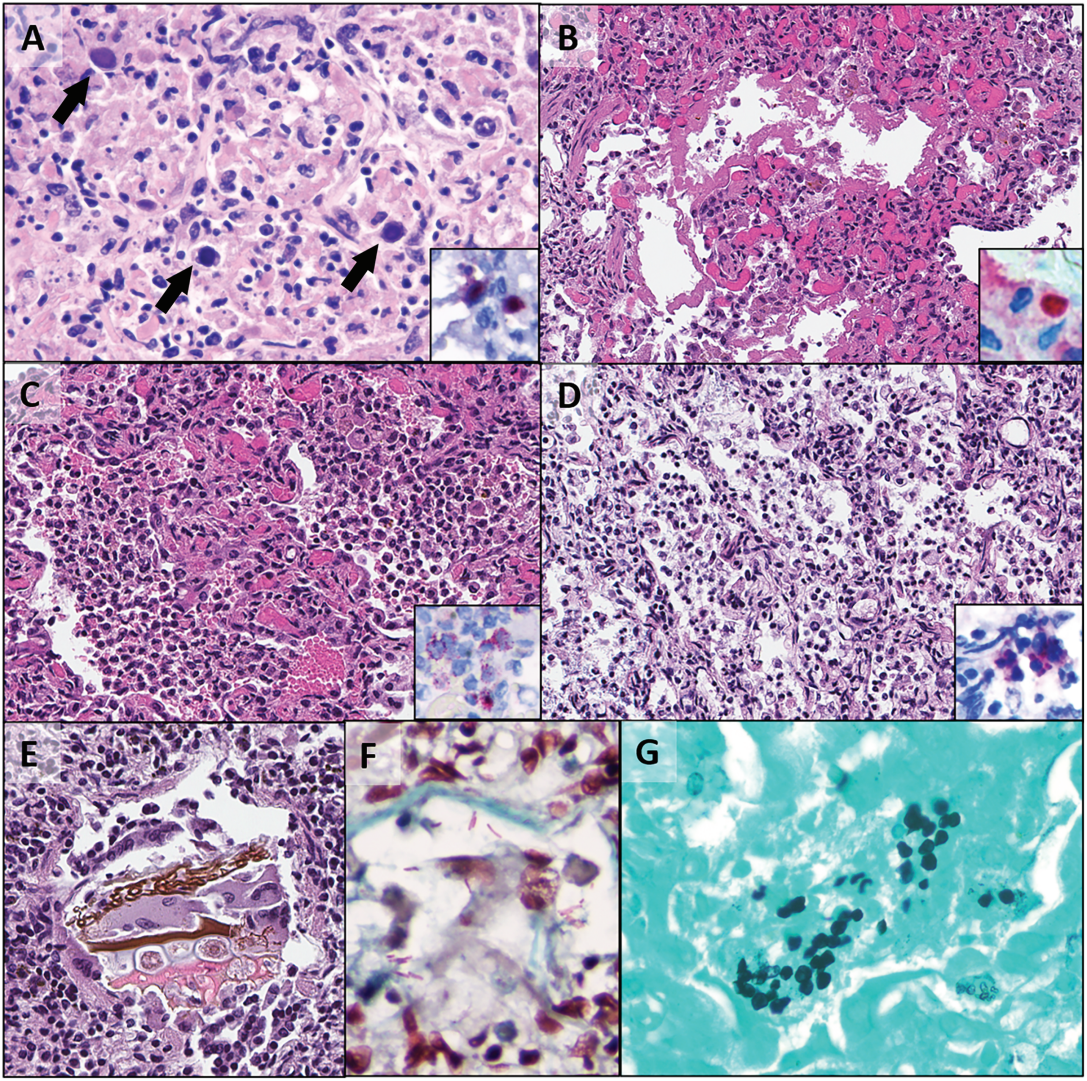
Pathology and identification of infectious agents by immunohistochemistry (IHC) or special stains in lung minimally invasive tissue sampling specimens. *A*, Necrotizing pneumonia with necrosis and viral inclusions (arrows); adenovirus IHC (inset); case 14. *B*, Interstitial pneumonitis and diffuse alveolar damage with hyaline membranes; parainfluenza virus 3 IHC (inset); case 2. *C*, Bronchopneumonia; *Streptococcus* spp IHC (inset); case 2. *D*, Bronchopneumonia; *Klebsiella* spp IHC (inset); case 15. *E*, Chronic aspiration with food material and giant cells; case 10. *F*, Acute aspiration with gram-negative bacterial rods; case 20. *G*, Grocott Methenamine Silver stain highlighting *Pneumocystis jirovecii*; case 6.

Overall case diagnoses were achieved utilizing all tests performed, and then correlation of TAC results with etiologic diagnoses were assessed. Assignment of etiologic diagnoses was based on the combination of relevant histopathologic findings and agent detection by 1 or more methods (Figure 2 and Supplementary Table 3). TAC contributed to etiologic agent detection in this context for 10 of 10 (100%) cases with etiologic agents identified in the lung, all for which the agent detected by TAC was confirmed by IHC or PCR (Figure 4). There were no cases with etiologic agents identified that were present on the TAC but not detected by it. Equivocal TAC results based on possibility of presence without overt pathology included CMV, human metapneumovirus (HMPV), coronavirus HKU1, and rhinovirus, and equivocal results based on consistent detection across multiple timepoints without confirmation by other methods included *Pneumocystis*, rhinovirus, *Moraxella*, and *Bordetella*. TAC results assessed as extraneous included adenovirus, *Pneumocystis*, *Staphylococcus, Streptococcus*, HMPV, respiratory syncytial virus, rhinovirus, *Moraxella*, *Haemophilus influenzae*, *Mycoplasma*, and *Klebsiella*; many of these were detected in cases with aspiration (Figure 2) and may be associated with carriage in the upper respiratory tract for those cases.

## Discussion

### Quantity of MITS Samples, but Not Number of Collection Timepoints, Affects Diagnostic Yield

Together with Dawa et al [16], we show that MITS is adequate for pathologic evaluation and molecular testing by TAC up to 72 hours after death, under conditions described in this study. A high quantity of samples increases the yield of identifying pathologic processes in MITS, irrespective of collection timepoint(s) over this postmortem interval. The value of more samples is related to the nature of the MITS procedure, which produces a single, small tissue fragment with each individual sampling. Viral infections and bacterial sepsis with widely distributed pathologic changes and infectious agents are more likely to be consistently detected than early or focal bacterial or fungal lesions with more limited tissue distribution. Accordingly, in this study, cases with etiologic viral agents detected by TAC had agent detection in an average of 9 of 10 lung specimens, while those with etiologic bacterial or fungal agents detected by TAC had agent detection in an average of 6 of 10 lung specimens.

### Pathology Drives Interpretation of TAC Results and Is Critical to Establishing an Etiologic Diagnosis in MITS

TAC is highly sensitive and indiscriminately detects nucleic acids from pathogenic, commensal, and contaminant microorganisms [13, 17]. Therefore, results must be interpreted together with relevant clinicoepidemiologic information, histopathologic findings, and any other laboratory results available. On one hand, TAC offers a more sensitive method of detection for some agents such as respiratory viruses that may otherwise be missed due to subtle or absent pathologic changes; on the other hand, bacterial agents in particular, but also some viral agents, may represent commensals or contaminants that confound overall case interpretation. This is especially true when there is aspiration with movement of upper airway commensals into the lower airways, which was common in this study (7/20 [35% of cases]) and in the authors’ experience with pediatric respiratory illness in general. Because multiple agents are often detected by TAC in cases of aspiration (up to 8 agents in 1 case with aspiration in this study), histopathology is imperative for differentiating aspiration from true polymicrobial infection.

For lung specimens without aspiration, viral contamination is less likely than bacterial contamination, and therefore more consideration is given to viral agents detected by TAC, while bacterial agents are more scrutinized as possible contaminants (eg, gastrointestinal or environmental). In this series, adenovirus and CMV in particular were detected by TAC in MITS specimens where overt evidence of viral cytopathic effect was not seen by histopathology. For cases where adenovirus was detected by TAC without viral cytopathic effect or IHC detection, adenovirus was either attributable to aspiration or was detected at few timepoints (<3) and in the context of another clear etiologic diagnosis; adenovirus was deemed extraneous in these cases. CMV was detected without viral cytopathic effect and was deemed equivocal in all cases with TAC detection except 1 (case number 9), where it was deemed extraneous due to TAC detection at a single timepoint and a clear alternative etiologic diagnosis (adenovirus). Bacterial agents and *Pneumocystis* were deemed contributory only if appropriate pathology and visual confirmation by IHC (or Grocott Methenamine Silver stain [GMS] for *Pneumocystis*) were present. Bacteria detected in absence of aspiration and for which IHC was negative or not available (eg, *Moraxella*, *Mycoplasma*) but detection by TAC occurred at multiple timepoints (eg, *Bordetella*) were deemed equivocal.

In addition to bacterial contamination of lung samples from the upper respiratory tract, lung and, more so, abdominal specimens (ie, liver) can be contaminated by gastrointestinal tissues that may be penetrated or inadvertently sampled during the MITS procedure, resulting in detection of gut flora by TAC. For this reason, TAC is generally not recommended on abdominal MITS specimens. Finally, in addition to overdetection of bacteria due to aforementioned reasons, underdetection may occur in the face of prior antibiotic treatment. Information regarding antibiotic administration was not available for the cases in this study.

Visualizing agents within pathologic lesions by histochemical or immunohistochemical stains markedly improves confidence in etiologic diagnoses. Traditional microbiologic methods such as blood and tissue cultures can also be performed on MITS specimens and help with interpretation of TAC, but are not without limitations, particularly related to contamination [14, 15, 18]. Ultimately, even with the use of multiple testing modalities and standardized, algorithmic interpretation of results, some degree of subjectivity and experiential bias remains in determining agent contribution and cause of death from MITS samples.

### Epidemiologic Value of MITS

Though not a specific goal of this study, several insights into the epidemiologic value of MITS were gleaned. First, this study unexpectedly identified a cluster of hospital-associated adenovirus infections, about which the clinical team was notified. Though complete assessment of cases in this study occurred after resolution of the outbreak in this instance, this finding demonstrates the potential utility of MITS and TAC for providing actionable information to improve health outcomes [13], even acutely in a localized outbreak. Other, larger-scale MITS studies have similarly identified nosocomial *Klebsiella* and multidrug-resistant *Acinetobacter baumannii* infections, the latter directly leading to implementation of improved infection control practices in that setting [19, 20]. Realizing the potential utility of MITS in outbreak situations, many MITS studies have very recently been either initiated or adapted to facilitate identification of severe acute respiratory syndrome coronavirus 2–related mortality during the ongoing coronavirus disease 2019 pandemic.

An initially puzzling finding in our study was the frequent and repeated detection of CMV, and to a lesser degree *Pneumocystis*, in multiple cases by TAC, without histopathologic or other test correlation. Because contamination was unlikely, these results were considered true positives but of equivocal or no etiologic significance, given the lack of pathology or detection in FFPE tissue. Despite the apparent clinical irrelevance of these agents among our cases, their detection by TAC heightens awareness to their presence and potential associated conditions and emphasizes the importance of understanding the geoepidemiology of pathogens where MITS is performed. CMV is ubiquitous worldwide, with higher prevalence and younger acquisition age in low-socioeconomic settings and developing countries [21]. One study in Kenya found an overall adult seroprevalence of 86%, with significant variability among different regions even within just this country [22]. The CMV seroprevalence rate in blood donors in Nairobi has been documented at 97% [23]. Congenital transmission, subclinical viremia, and prolonged latency with intermittent reactivation, especially with immunosuppression, are integral to maintaining its high prevalence [21, 22]. Viremia or latency in lung without active disease may have resulted in TAC detection of CMV in our study cohort [24].

*Pneumocystis* was also detected by TAC in multiple cases without histopathologic or other test correlation. Similar to CMV, its detection without established significance nevertheless piques concern for immunosuppressive conditions, including malnutrition and HIV infection, both of which are historically associated with *Pneumocystis* pneumonia [25-27], and both of which are relatively common in our study’s geographic region. Three case patients in our study had clinically documented malnutrition, and 2 others had severe hepatic steatosis compatible with malnutrition. One of these patients (case number 6) had both CMV and *Pneumocystis* detected by TAC and was the only case to have *Pneumocystis* identified by GMS stain. These findings, together with observed lymphoplasmacytic interstitial pneumonitis, prompted retrospective testing of stored blood specimens for HIV, which was positive. This case, and its pathway to HIV diagnosis, emphasizes the importance of understanding the interrelatedness of clinicopathologic, epidemiologic, and pathogenetic factors when evaluating MITS specimens and TAC results obtained from them, and highlights the potential for MITS in leading to relevant diagnoses even sometimes outside of specific, targeted testing. Conversely, it also demonstrates that even TAC results deemed “extraneous” in terms of contribution to death may still yield important information toward overall case evaluations in MITS.

### Limitations

One major limitation of comparing TAC and other results obtained from MITS is that samples evaluated by TAC and those evaluated by histopathology and FFPE-based testing methods are not represented by the exact same sample collected at any 1 timepoint. During the MITS procedure, separate needle core samples are collected for TAC vs histopathologic evaluation and other testing. Due to the nature of this collection, it is conceivable that the 2 samples being compared by TAC and other methods have different pathologic features and pathogen presence, reducing correlation between TAC and other results for individual samples. This accounts for the lower agreement observed between TAC and FFPE test methods (IHC or PCR) (43%), as compared to agreement between IHC and PCR (69%), which are performed on the same FFPE tissue block. This limitation can be minimized by increasing the overall number of samples obtained and interpreting results on a case-level basis for all test types in aggregate, rather than comparing individual samples. It also highlights the importance of considering results from all various diagnostic methods together when assigning infectious etiologies, rather than relying on one particular diagnostic method in MITS. This study utilized a state-of-the-art laboratory, enabling thorough workup of cases by multiple diagnostic methods to reach etiologic diagnoses. Such rigorous testing is not feasible in many settings where MITS is performed, which further elevates the importance of clinicopathologic correlation with TAC.

Another limitation is a lack of unrefrigerated bodies for result comparison with those kept under refrigeration in this study. The stark lack of autolysis and postmortem bacterial translocation/overgrowth was unexpected, and while lending to “cleaner” results, may not be representative of TAC findings in routine, real-world scenarios without refrigeration. Detection of more agents would be expected to confound interpretation of results in those situations. Regardless, these findings are useful and can serve as baseline for comparison with future studies using unrefrigerated samples. Finally, all cases enrolled in this study had clinical diagnoses of respiratory illness, which biased toward detection of respiratory pathogens by TAC and other test methods. When MITS is used on a larger scale for general mortality and unexplained death surveillance, the diagnostic yield may not be as high.

## Conclusions

MITS, coupled with both traditional and advanced diagnostic methods, provides an opportunity to ascertain causes of death and to detect infectious etiologies in cases where CDA is not possible. TAC is a powerful method for rapid detection of many pathogens in MITS specimens, but interpretation of results can be confounded by detection of extraneous microorganisms or those that are truly present but may not be associated with active disease. TAC is more sensitive than histopathology or FFPE-based test methods (IHC and PCR) alone and is particularly useful for detection of viruses, while traditional pathology methods are critical for accurately identifying bacterial etiologies of significance, especially when aspiration is present. Regardless of testing methods used, adequate tissue sampling and contextualization of laboratory results with histopathologic findings and any available clinicoepidemiologic data are of utmost importance when ascribing causes of death based on MITS.

## Supplementary Data

Supplementary materials are available at *Clinical Infectious Diseases* online. Consisting of data provided by the authors to benefit the reader, the posted materials are not copyedited and are the sole responsibility of the authors, so questions or comments should be addressed to the corresponding author.

ciab772_suppl_Supplementary_Materials

## Notes

***Acknowledgments.*** The authors thank the following individuals for their technical support: the Kenyatta National Hospital MITS study team, including Washington Ochieng, Walter Barake, John Mathaiya, Cyrus Waciury, John Muriuki, Abdulaziz Baya, and Peter Ouma; Nancy Otieno (Kenya Medical Research Institute); Marc-Alain Widdowson (Division of Global Health Protection, Centers for Disease Control and Prevention [CDC], Nairobi, Kenya); John Mathaiya (Thika Level V Hospital, Kiambu County, Kenya); Peter Muturi and Milka Bunei (Washington State University, Global Health Programs, Kenya); Kathy Banke and Scott Dowell (Bill & Melinda Gates Foundation); Cynthia Whitney (Emory University, Atlanta, Georgia), and Dianna Blau and Jennifer Verani (CDC). The authors also express gratitude to the staff of the Kenyatta National Hospital in Nairobi who supported the logistics of this study, and to the families of the deceased children for agreeing to their child’s enrollment in the study.

***Disclaimer.*** The findings and conclusions herein are those of the authors and do not necessarily represent the official position of the CDC. The mention of company names or products does not constitute endorsement by the CDC. The funders did not play a role in study design, data interpretation, or the decision to submit the work for publication. Under the grant conditions of the Bill & Melinda Gates Foundation, a Creative Commons Attribution 4.0 Generic License has already been assigned to the author-accepted manuscript version that might arise from this submission.

***Supplement sponsorship.*** This supplement is sponsored by MITS Surveillance Alliance Secretariat, led by RTI International, with funding from the Bill and Melinda Gates Foundation.

***Financial support.*** This work was supported by the Bill & Melinda Gates Foundation (opportunity/contract ID OPP1168515).

***Potential conflicts of interest.*** E. W. has received grants from the MITS Alliance and Institute bioMérieux and has received financial support from Roche PLC. M. D. and J. W. hold the following patents/applications: US patent 10.072.305 (issued 2018); PCT application number PCT/US2013/028034 (filed 2013); US application number 14/398390 (filed 2014); US application number 16/056147 (filed 2018). All other authors report no potential conflicts of interest.
