# Supplementary material for: Histopathology Is Key to Interpreting Multiplex Molecular Test Results From Postmortem Minimally Invasive Tissue Samples
Source: Clin Infect Dis. 2021 Dec 15;73(Suppl 5):S351–9. doi: 10.1093/cid/ciab772 (PMC8672755; doi:10.1093/cid/ciab772)
Supplement: ciab772_suppl_Supplementary_Materials [file ciab772_suppl_supplementary_materials.docx]

**SUPPLEMENTARY MATERIALS**

**Histopathology is key to interpreting multiplex molecular test results from postmortem minimally invasive tissue samples (MITS)**

METHODS………………………………………………………………………………………………………………………… 2

Histology………………………………………………………………………………………………………………. 2

Immunohistochemistry…………………………………………………………………………………………. 2

FFPE PCR……………………………………………………………………………………………………………….. 3

Assessment of MITS adequacy and quality……………………………………………………………..4

SUPPLEMENTARY TABLES……………………………………………………………………………………………………5

Supplementary Table 1……………………………………………………………………………………………5

Supplementary Table 2………………………………………………………………………………………..….7

Supplementary Table 3……………………………………………………………………………………………8

REFERENCES……………………………………………………………………………………………………………………….10

**METHODS**

***Histology***

Samples for histopathology were formalin fixed and routinely processed for paraffin histology, sectioned and stained with hematoxylin-eosin (H&E) and Lillie-Twort Gram stains. Histopathologic evaluation was performed independently by two or three pathologists (RM, EW, JR) for each case, and interpretation of findings and final diagnoses were reached by consensus. Grocott methenamine silver (GMS) stain for *Pneumocystis*, immunohistochemistry (IHC) and/or PCR testing for other specific infectious agents was performed on the formalin-fixed paraffin-embedded (FFPE) tissues at the pathologists’ discretion, based on findings, and was conducted in general accordance with the testing algorithm previously described by the authors [1]. For purposes of correlating tests results, GMS results for Pneumocystis were considered equivalent to IHC results for other agents.

***Immunohistochemistry***

Immunohistochemical assays were performed using an indirect immuno‐alkaline phosphatase detection methodology [1]. Four micron tissue sections were deparaffinized and rehydrated through graded alcohol before staining. Tissue samples were pretreated with Proteinase K (Roche, Indianapolis, IN, USA) or heat-induced epitope retrieval with a citrate-based buffer (Biocare Medical, Concord, CA, USA), according to a standardized protocol (Supplementary Table 2). After blocking with Background Punisher (Biocare Medical), the slides were incubated with the primary antibody (Supplementary Table 2). Colorimetric detection of linked antibodies was performed using the Mach 4 AP Polymer kit (Biocare Medical, Concord, CA, USA), followed by visualization with Permanent Red Chromogen (Cell Marque/Millipore Sigma, Rocklin, CA). Positive controls containing infected cells or tissue samples from a previously confirmed case were run in parallel. Slides were counterstained with Mayer's hematoxylin (Polyscientific, Bay Shore, NY, USA), blued in lithium carbonate (Polysciences, Inc., Warrington, PA, USA), and then coverslipped with aqueous mounting medium (Polysciences, Inc.).

***FFPE PCR***

RNA was extracted from FFPE tissue specimens using the EZ1 RNA Tissue Mini Kit on EZ1 Advanced XL (Qiagen) as described previously [2]. Real-time RT-PCR assays for influenza A and B viruses, human parainfluenza viruses (HPIV) types 1–4, respiratory syncytial virus (RSV), human metapneumovirus (hMPV) and rhinovirus were performed using previously published primers and probes [2-5] and the Invitrogen Superscript III Platinum One-Step qRT-PCR Kit (ThermoFisher Scientific) on the Stratagene Mx3005P QPCR System (Agilent Technologies). B2-microglobulin was used as an internal control to ensure quality of RNA extraction [2].

DNA was extracted from FFPE tissue specimens using the QIAamp Ultra Clean Production (UCP) Pathogen DNA Mini Kit (Qiagen) and evaluated by a conventional PCR assay for cytomegalovirus (CMV), adenovirus and *Mycoplasma pneumoniae* using previously published primers [6-8]. PCR positive amplicons were identified by gel electrophoresis, extracted from the gel, and directly sequenced by Sanger sequencing on a GenomeLab GeXP sequencer (AB SCIEX LLC, Redwood City, CA, USA). The search for homologies to known sequences was performed by using the BLAST nucleotide database (http://blast.ncbi.nlm.nih.gov/Blast.cgi). The presence of amplifiable DNA in all extracts was verified by amplification of human house-keeping genes beta-globin and glyceraldehyde-3-phosphate dehydrogenase.

For Streptococcal PCR assays, tissue scrolls underwent Qiagen pretreatment for paraffin-embedded tissue, followed by 200 µL of ATL Buffer and incubated at 56^o^C overnight. The dried pellet was treated with 20 µl of proteinase K at 56^o^C for 30 minutes. The solution was transferred (up to 400 µL) to an individual MagnaPure sample tube with 300 µL of Buffer #4 Isolation Kit III (bacteria, fungi) and proceeded using the external lysis protocol according to manufacture instructions [9]. DNA extracts were eluted to 100 µL and stored at -20^o^C until PCR testing was performed using Quanta Biosciences PerfeCTa® qPCR ToughMix®, Low ROX™, for *S. pneumoniae* - *lyt*A gene [10], *S. agalactiae* – *cfb* gene [11], *S. pyogenes* - *spy* gene [12] and PerfeCTa Multiplex qPCR ToughMix®, Low ROX™, and for the pneumococcal multiplex serotyping assays [13].

***Assessment of MITS adequacy and quality***

MITS samples from each collection were assessed for target tissue adequacy, postmortem autolysis, and presence of postmortem bacteria. Collection was adequate if two or more core fragments of the target tissue type were present. Non-target tissues collected were also documented. Autolysis was evaluated qualitatively based on alterations in overall tissue architecture, cellular features, and difficulty detecting lesions [14, 15]. Presence of bacteria was assessed by Gram stains; perimortem aspiration and postmortem translocation/overgrowth was considered for bacteria seen in absence of tissue alterations indicating a pathologic bacterial process.

**SUPPLEMENTARY TABLES**

**Supplementary Table 1. Agents tested by blood and respiratory TAC**

| **Pathogen** | **Blood TAC card** | **Lung TAC card** |
| --- | --- | --- |
| Acinetobacter baumanii |  | x |
| Adenovirus | x | x |
| B. parapertussis, B. bronchiseptica |  | x |
| Bordetella spp (B. pertussis or B. holmseii) |  | x |
| Burkholderia pseudomallei |  | x |
| Chikungunya virus | x |  |
| Chlamydia pneumoniae |  | x |
| Chlamydia trachomatis |  | x |
| Corynebacterium diphtheriae |  | x |
| Corynebacterium pseudotuberculosis |  | x |
| Corynebacterium spp. (tox gene) |  | x |
| Cryptococcus neoformans, Cryptococcus gattii | x |  |
| Cytomegalovirus |  | x |
| Dengue virus | x |  |
| Enterovirus | x | x |
| Escharichia coli/Shigella | x |  |
| Group A Streptococcus | x | x |
| Group B Streptococcus | x | x |
| Haemophilus influenza | x | x |
| Haemophilus influenzae type B | x |  |
| Human coronavirus 229E |  | x |
| Human coronavirus NL63 |  | x |
| Human coronavirus OC43 |  | x |
| Human coronavirus HKU1 |  | x |
| Human metapneumovirus |  | x |
| Influenza A |  | x |
| Influenza B |  | x |
| Klebsiella pneumoniae | x | x |
| Listeria monocytogenes | x |  |
| Measles |  | x |
| MERS coronavirus (N2 gene) |  | x |
| MERS coronavirus (upE gene) |  | x |
| Moraxella catarrhalis |  | x |
| Mycobacterium pneumoniae |  | x |
| Mycoplasma pneumoniae |  | x |
| Neisseria meningitides | x |  |
| Orientia tsutsugamushi | x |  |
| Parainfluenza virus type 1 |  | x |
| Parainfluenza virus type 2 |  | x |
| Parainfluenza virus type 3 |  | x |
| Parainfluenza virus type 4 |  | x |
| Parenchovirus | x |  |
| Pertussis toxin - B. parapertussis or B. pertussis |  | x |
| Plasmodium falciparum | x |  |
| Plasmodium vivax | x |  |
| Pneumocystis jirovecii |  | x |
| Pseudomonas aeruginosa | x | x |
| Respiratory syncytial virus |  | x |
| Rhinovirus |  | x |
| Rickettsiae species | x |  |
| Rubella virus | x | x |
| Salmonella enterica Typhi | x |  |
| Salmonella paratyphi A | x |  |
| Salmonella species | x |  |
| Staphylococcus aureus | x | x |
| Streptococcus pneumoniae | x | x |
| Toxoplasma gondii | x |  |
| Treponema pallidum | x |  |
| Varicella zoster virus |  | x |
| Zika virus | x |  |

**Supplementary Table 2. Antibodies and conditions used for immunohistochemistry in lung MITS samples**

PK: proteinase K; AR: antigen retrieval, citrate buffer

**Supplementary Table 3. Case level agreement of TAC, IHC, and PCR results**

N/A: Not available**;** NEG: Negative

^a^ For purposes of correlating tests results, GMS results for Pneumocystis were considered equivalent to IHC results for other agents

^b^ Streptococcus spp. identified by IHC may not be present on TAC card, not included in totals

^c^ Only one FFPE method (IHC or PCR) available, not included in totals

**REFERENCES**

1. Martines RB, Ritter JM, Gary J, et al. Pathology and telepathology methods in the Child Health and Mortality Prevention Surveillance network. Clin Infect Dis, **2019**; 69(Suppl 4): S322-S332.
2. Denison AM, Blau DM, Jost HA, et al. Diagnosis of influenza from respiratory autopsy tissues: Detection of virus by real-time reverse transcription-PCR in 222 cases. J Mol Diagn, **2011;** 13: 123-8.
3. Weinberg GA, Schnabel KC, Erdmann DD, et al. Field evaluation of Taqman Array Card (TAC) for the simultaneous detection of multiple respiratory viruses in children with acute respiratory infection. J Clin Virol, **2013**; 57: 254-60.
4. Kim C, Ahmed JA, Eidex RB, et al. Comparison of nasopharyngeal and oropharyngeal swabs for the diagnosis of eight respiratory viruses by real-time reverse transcription-PCR assays. PLoS One, **2011**; 6(6): e21610.
5. Lu X, Holloway B, Dare RK, et al. Real-time reverse transcription-PCR assay for comprehensive detection of human rhinoviruses. J Clin Microbiol, **2008**; 46(2): 533-9.
6. Lu X, Erdman DD. Molecular typing of human adenoviruses by PCR and sequencing of a partial region of the hexon gene. Arch Virol, **2006**; 151(8): 1587-1602.
7. Gunson RN, Maclean AR, Shepherd SJ, Carman WF. Simultaneous detection and quantitation of cytomegalovirus, Epstein-Barr virus, and adenovirus by use of real-time PCR and pooled standards. J Clin Microbiol, **2009**; 47(3): 765-70.
8. Morozumi M, Nakayama E, Iwata W, et al. Simultaneous detection of pathogens in clinical samles from patients with community-acquired pneumonia by real-time PCR with pathogen-specific molecular beacon probes. J Clin Microbiol, **2006**; 44(4): 1440-6.
9. DNeasy Blood and Tissue Handbook, Quiagen, 2020.
10. Carvalho Mda G, Tondella ML, McCaustland K, et al. Evaluation and improvement of real-time PCR assays targeting lytA, ply, and psaA genes for detection of pneumococcal DNA. J Clin Microbiol, **2007**; 45: 2460-6.
11. Diaz MH, Waller JL, Napoliello RA, et al. Optimization of multiple pathogen detection using the TaqMan Array Card: Application for a population-based study of neonatal infection. PloSOne, **2013**; 8(6): e66183.
12. Kodani M, Yang G, Conklin LM, et al. Application of TaqMan low-density arrays for simultaneous detection of multiple respiratory pathogens. J Clin Microbiol, **2011**; 49(6): 2175-82.
13. Pimenta FC, Roundtree A, Soysal A, et al. Sequential triplex real-time PCR assay for detecting 21 pneumococcal capsular serotypes that account for a high global disease burden. J Clin Microbiol, **2013**; 51: 647-52.
14. Lesnikova I, Schreckenback MN, Kristensen MP, Papanikolaou LL, Hamilton-Dutoit S. Usability of Immunohistochemistry in Forensic Samples With Varying Decomposition. Am J Forensic Med Pathol, **2018**; 39(3): 185-191.
15. George J, Van Wettere AJ, Michaels BB, Crain D, Lewbart GA. Histopathologic evaluation of postmortem autolytic changes in bluegill (Lepomis macrohirus) and crappie (Pomoxis anularis) at varied time intervals and storage temperatures. PeerJ, **2016**; 4: e1943.
